# Keeping young researchers out of harm’s way: conducting youth participatory action research with young people experiencing homelessness

**DOI:** 10.3389/fpubh.2024.1386714

**Published:** 2024-07-03

**Authors:** G. Allen Ratliff, Darren Cosgrove, Jessica O. Yang, Richard Sarabia, Taylor L. Harvey, Nathan Jeffcoat, Marguerita Lightfoot, Sherilyn Adams, Ilsa Lund, Colette L. Auerswald

**Affiliations:** ^1^School of Social Work, University of Nevada, Reno, Reno, NV, United States; ^2^School of Social and Behavioral Sciences, Marist College, Poughkeepsie, NY, United States; ^3^Mailman School of Public Health, Columbia University, New York, NY, United States; ^4^School of Public Health, University of California, Berkeley, Berkeley, CA, United States; ^5^OHSU-PSU School of Public Health, Portland, OR, United States; ^6^Larkin Street Youth Services, San Francisco, CA, United States

**Keywords:** participatory action research, homelessness, qualitative, community-based research, youth

## Abstract

**Introduction:**

Youth Participatory Action Research (YPAR) is an approach to conducting research with youth populations in order to effectively engage youth in research that impacts their lives. Young people experiencing homelessness (YEH) are vulnerable to power and social environments in ways that call attention to their experiences in research.

**Methods:**

The context for this paper was a qualitative YPAR project to incorporate youth voice into the operations of a larger research study that hired youth as researchers. Participant-researchers provided feedback and consultation with senior staff in order to improve their access to resources, safety, and stability.

**Results:**

Themes that emerged from thematic analysis of reflections, discussions, and meetings showed the need for consistent access to food, the risk of environmental violence targeting youth researchers, the structural and experiential barriers to professional engagement, and the benefits that young researchers experienced as part of their work in the study.

**Discussion:**

Recommendations and lessons learned are described, notably to ensure that youth are paid and provided food, to construct effective safety plans during fieldwork, and to provide a flexible, inclusive, trauma-responsive approach to supervision of project tasks.

## Introduction

Research methodologies that focus on collaborative partnerships with the target population in order to redress inequities in the research setting have long been used in critical and empowerment-focused scholarship (1, 2). Such approaches, including participatory action research, youth participatory action research, and community-based participatory research, are meaningful approaches to knowledge production in partnership with communities who experience social and political marginalization (1, 3). Participatory action research (PAR) takes a critical approach to scientific inquiry in its attempt to utilize data collection and analysis methods that advance leadership and skills among participants, while also engaging in tangible action that is both informative and socially transformative (3, 4). Youth-focused PAR, or youth participatory action research (YPAR), is an approach to participant-led research that centers the voice, agency, and leadership of the young people it engages as means to promote transformative action (3). As an approach and not a method itself (e.g., surveys, focus groups, arts-based research, etc.), YPAR is focused on how methods and tools can be applied in such a way that youth and adult researchers share power and young people’s lived experiences are given priority (5).

Many researchers working in or with communities, or those studying social problems stemming from systemic oppression, utilize participatory methods in an effort to avoid and challenge the harms they perceive other more “traditional” methods to inflict upon already marginalized communities (6, 7). Tensions related to power and competing priorities may raise ethical dilemmas among collaborative research teams (8, 9). For example, YPAR projects funded or initiated by an organization or institution (e.g., a university or college) structurally provide more power to the institutionally-affiliated researcher, who often is the person organizing and controlling the funds for the project [see (8)]. Therefore, while the “lead” researcher may strive to construct processes and procedures that are egalitarian and democratic in nature, it is the researcher who is choosing to share power with youth participants (8). Dilemmas related to power sharing may result in some cases in youth being tokenized, wherein, the project claims participatory leadership and to represent youth voice, but in practice shares little real leadership. Moreover, questions regarding consent, risk-management, representation, compensation for time, and authorship of works produced from the research are additional ethical considerations that must be examined (8, 10, 11).

### Youth experiencing homelessness

Youth homelessness is an issue of national importance in the United States (US). According to the 2017 Voices of Youth Count, an estimated 4.2 million youth and young adults (18–25) experience homelessness in the US. This translates to one in 10 young adults experiencing homelessness within a single calendar year in the US, with half this number accounted for by youth who are couch surfing (12, 13). Risk factors for youth homelessness include difficulties within familial relationships, mental health or substance use issues, poor schooling history, history of foster care, homelessness as a child, and running away from home (12–15). Young people who identify as LGBTQIA+ are at particular risk of homelessness (16, 17). Homelessness and its comorbidities can present obstacles to the learning and growth necessary for proper development during adolescence and young adult developmental periods (18, 19). This can result in physical and mental illnesses, experiences of violence, unintended pregnancy, premature exit from education, substance use, and even early death (14, 15, 20, 21). In order to prevent and mitigate these negative outcomes, engaging with YEH themselves in the research process is critical (22, 23).

Youth experiencing homelessness (YEH) have historically participated in research as subjects rather than as researchers. Studies that do involve YEH participation are largely photovoice studies, but the magnitude of YEH participation in these studies can vary greatly (24, 25). Foundationally, YPAR recognizes “youth” as an age group whose disenfranchisement is enabled by adult dismissal of their agency and personhood (2, 26). YPAR challenges this disenfranchisement by enabling youth critiques of traditional research methodology and centering youth in research leadership (2). When utilized with YEH, YPAR has the potential to create new opportunities for improving services and health outcomes in this population.

Given the philosophical and practical implications of YPAR research and the unique vulnerabilities that YEH face in their social environments, it is important to consider how research can be conducted with YEH in order to avoid further harm or limitations on their ability to participate in research. There is a growing body of work demonstrating the benefits and empowerment of YPAR with YEH (2, 23, 27), yet there remains little research on how YPAR can be best applied with vulnerable youth populations or the practical needs of conducting research with vulnerable youth as researchers. Aviles and Grigalunas (28) suggest the need for flexible and inclusive work contexts for YEH researchers, as well as an emphasis on providing safe spaces for youth to conduct their work. In reporting a YPAR study with YEH, Robson et al. (29) describe instability in the lives of YEH as a barrier for participation in research activities and suggest the need for stronger connections with YEH-serving organizations during the course of YPAR with YEH.

YPAR has received increased attention as an effective research approach that draws on the expertise of young people to understand and improve the circumstances that affect their lives. YEH are young people who are deeply affected by the circumstances of their lives, the social systems through which they attempt to navigate the world, and the social environments and cultures that directly impact them without asking for their input or feedback. The study described here was part of a larger study on youth homelessness that utilized a YPAR approach to examine the experiences of YEH in finding safety, violence, and accessing resources in an urban setting. As part of the YPAR approach, the youth researchers who implemented the study were included as a separate population of research participants to examine how to effectively conduct YPAR with this population. The data, findings, and implications reported here address the needs of youth researchers engaged in YPAR work in their own communities. The aim is to present the lessons learned to guide future researchers in implementing ethical, trauma-responsive, and empowering YPAR with vulnerable youth populations.

## Methods

The findings reported here emerged from a larger study that utilized a YPAR approach to engage in “walking tours” and Participatory Photomapping (30, 31) as a method of collecting data on YEH experiences and expertise regarding the places and spaces of violence, safety, and resources. Walking tours and Participatory Photomapping are data collection methods that engage research participants actively in the physical places where they are describing their experiences (30, 31). In the larger study, participants walked with youth researchers during interviews that occurred in the neighborhoods where the participants spent their time.

The study implemented a YPAR approach by connecting with community convenings of YEH within the local area and offering to partner with youth to conduct research that asked and tried to answer the questions that YEH have about their lives. YEH in these convenings felt strongly that research needed to be done on how they experience violence and navigate physical spaces across the city to find resources. The principal investigators then designed a study under the ongoing consultation and supervision of YEH at these convenings; this ongoing relationship was maintained throughout the life of the study.

The principal investigators included two senior researchers from separate institutions and the executive director of a community-based social service agency serving YEH. The study leadership team further included: (1) a study logistical coordinator who was the director of strategy for the community-based partner agency, and (2) a project director/doctoral student who supervised the young researcher team and day-to-day study operations. The larger project engaged youth exiting homelessness or with lived experience of homelessness and young people with service experiences in homelessness as community informants and then as researchers as part of the YPAR approach to the study. The young researchers were hired and paid as staff of the community-based YEH-serving social service agency; trained in research methods, ethics, and interviewing; supervised as they conducted data collection in the field; and then trained and guided through the data analysis process for the study. More details on the wider project and findings that have emerged from this study can be found at Ratliff et al. (32) and Tan et al. (33). The findings reported here focus on the process of conducting a YPAR study with a vulnerable population of youth researchers and present data obtained from the youth researchers themselves, not the YEH research subjects they interviewed in the main study, along with other data from study team members related to the experiences and decision-making in implementing the larger study with a team of youth researchers.

### Participants: youth researchers

The youth researcher team (i.e., the “participants” of the study findings reported here) was composed of six youth, three young people who were “exiting” homelessness (i.e., participating in some form of transitional or permanent supportive housing program) and who had experienced homelessness within the previous 2 years, and three young people who were undergraduate students with lived experience of homelessness and/or service experience with housing and homelessness. Youth researchers were recruited from two groups (YEH and students) in an attempt to bring diverse skills and capacities to the project and build community between youth researcher colleagues. Youth researchers applied for the roles after announcements were made at YEH convenings and in agencies serving YEH. Youth researchers were interviewed by the project director and all applicants were offered positions on the team (two declined due to accepting other employment). The initial composition of the youth research team included four students and six young people exiting homelessness. Four youth researchers exited the project, two prior to data collection, including one student and one YEH, and two more exited during data collection, both of which were YEH. The youth researchers were hired as agency staff, paid hourly wages above minimum wage, and were supervised and trained as staff. The core team of six youth researchers conducted the entirety of data collection and majority of data analysis for the broader study. The initial and core youth researcher team members were of diverse racial, sexual, and gender identities. All six core youth researchers were provided an invitation for authorship and four members of this team of youth researchers opted to participate as authors (the other two declined due to lack of time and interest). Youth researchers provided informed consent to allow for assessment of the YPAR study. Approval was obtained from the Institutional Review Board of the University of California, Berkeley.

### Data collection

The data presented here are qualitative data collected over the course of the study as reflections, observations, and exit interviews, from May 2018 to June 2019, completed by the youth researchers and the project director. The data include: (1) reflections completed by the youth researchers during weekly team meetings, (2) supervision notes from the project director over the course of regular one-on-one mentorship and supervision meetings with youth researchers and in consultation with the senior research team, (3) evaluations of training and field operations during the data collection phase of the study, and (4) the transcripts of semi-structured exit interviews conducted with the youth researchers after data analysis was completed. Reflections occurred consistently during weekly team meetings in which youth researchers were asked about what they learned, what they felt confident about, what they needed, and what they suggested. Individual supervision occurred with the project director and each youth researcher approximately once every other week during data collection. Semi-structured exit interviews with each youth researcher individually occurred after data collection and data analysis were completed and focused on youth researcher experiences in working on the project as part of the study team. Interviews were conducted by a graduate student in public health who was not otherwise affiliated with the project.

### Data analysis

These data were compiled, reviewed, and analyzed using thematic analysis (34) by members of the author team, including youth researchers, to describe their experiences and identify key challenges and opportunities that arose during the course of the YPAR study. During the course of the study, the project director held weekly meetings and daily debriefing sessions with the youth researchers, where experiences were identified and discussed. The senior researchers also met weekly and reviewed reflections and supervision notes, in addition to discussing the ongoing needs, challenges, and successes of the study. Exit interview transcripts were deidentified and a summary was provided by the otherwise-unaffiliated graduate student, then these deidentified transcripts and summaries were reviewed by the senior research team and youth researchers to identify key themes that emerged in the interviews. The analyses of live data (reflections, supervision, discussions) were iterative throughout the course of the study, considering themes that emerged from the youth researchers and the study investigator experiences. The analyses of exit interview transcripts were completed through a review by the study investigators who then discussed and described central themes in a focused analytic review of the data following the completion of data collection and analysis. Youth researchers engaged in discussion to review key themes and provide additional context and nuance to the thematic analyses.

Descriptive codes and theme identification developed over time and in consultation with study team members as the study progressed. Foundational patterns that emerged into the themes presented were discussed openly during team meetings with youth researchers and senior researchers, recognizing how these patterns were noticed and impacted the youth researchers (34). Youth researchers supported the articulation of these themes in preparation of the report of these findings, with consensus easily reached across the study team upon completion of the outline of the findings. Data presented here were reviewed individually by the first, second, and third authors, then discussed as an author team for confirmation. The youth researchers are co-authors, and have reviewed and approved every version of this manuscript prior to submission.

## Results

Five major themes emerged through analysis of the data: (1) food insecurity; (2) environmental violence; (3) structural barriers to consistent participation in employment, such as legal issues and documentation requirements; (4) limited job and life skills training and consistency that resulted in difficulties with professional behavior and expectations; and (5) youth researcher pride and satisfaction with their participation. The challenges and opportunities of each theme are presented along with the ways in which they were navigated by the senior research team and the youth research team.

### Food insecurity

Food insecurity emerged as an early concern. Youth researchers, especially those who were exiting homelessness, would arrive hungry to their work shifts during training and data collection. Youth researchers were being paid, but the high cost of living in the area and the delay in receiving paychecks due to normal payment processes (i.e., paychecks were issued 2 weeks after each time period when timesheets were due) meant that they did not receive their paychecks for several weeks after starting. Furthermore, much of their pay went to cover the cost of rent and other necessities.

The study team addressed this issue by shifting resources to ensure youth researchers were provided at least two meals on every day that they worked on the project. This study was embedded in the youth-serving community agency employing the young researchers. The agency leaders on the senior research team ensured that youth researchers could access the daily lunches provided to agency clients. The agency further covered the cost of a second meal at the end of each work shift. This required substantial coordination between the study leadership and agency leadership and staff to ensure that the young researchers were able to access the lunches despite not being agency clients, and to support changes to the agency’s study budget to include additional food. Communication between agency staff and research staff was necessary to ensuring that the youth researchers had consistent, adequate access to food on the days that they worked. One of the young researchers said in an exit interview: “One thing that was really helpful was having food, just making sure that people got something to eat, and were actually able to function.” Another of the young researchers said: “[The project director was] always making sure that we were taken care of, with food and water and taking breaks. And checking up on us personally, I feel like [they] really went above and beyond to meet with us. I was going through a lot with my housing and stuff. And for [them] to do that, to have your boss actually care, (getting emotional), it was just—that was a lot.”

### Environmental violence

Young researchers, especially queer- and female-identified researchers, encountered multiple episodes of environmental harassment and threats of violence during their street-based research, despite careful protocols designed to maximize team safety. The field team (project director and young researchers) would move together from the partner agency site to the planned project sites for each day, usually by way of public transportation (e.g., trains, buses, trams). While the youth researchers were recruiting participants and conducting the walking tour interviews, the project director would be stationed nearby (usually at a local coffee shop), staying in touch with youth via walkie-talkies and mobile phones. Youth interview teams were composed of three researchers, one conducting the interviews, one managing the tablet and recording devices, and a third who was the “sentinel,” whose role was to maintain constant observation of the environment and navigate the team away from potential risks as they moved through the city. Given the social-environmental vulnerability of youth and YEH, young researchers were subject to harassment and threats from outside entities during transit (even while moving as a group) and during the walking tour interviews. There were numerous instances of ‘catcalling’ and other misogynistic, racist, and homo−/transphobic harassment from a variety of sources. One example occurred on a bus, when a young woman researcher was verbally accosted by a man who then threw water at her. In another instance, a bottle was thrown at a young woman researcher, who reacted quickly to dodge the projectile. The queer and transgender young researchers were harassed and followed at times, including an instance when a study interview team ducked into an open shop to avoid a confrontation during an interview. One young researcher said: “I felt like sometimes my specific presented identities made my experience less safe, or clashed with the needs of the day in ways I could not avoid.”

Methods to increase the safety of the youth researchers were implemented by the research team, before and during the start of the data collection phase of the study. The initial training period included multiple discussions on safety and situational awareness, creation of safety protocols, and practice in moving as a group through the streets. After the data collection phase began, further safety protocols were implemented. First, additional interview protocols were developed to complete walking tour interviews virtually, using a tablet with geospatial surveying software. This allowed the team to complete interviews in neighborhoods or environments that felt unsafe to the youth researchers and/or the youth participants of the study. Second, the team developed clear signals to each other and the project director to communicate if they were feeling unsafe. The project director empowered youth researchers to handle situations themselves, recognizing their own strengths in such situations, and would actively interpose when signaled, directing the attention of aggressors toward them and away from the young researchers. This approach provided youth researchers with opportunities to hone their interpersonal and deescalation skills and utilize their strengths and lived expertise while knowing that backup was nearby. For example, the young woman researcher responded to the verbal and watery assault on the bus by forcefully swearing at the perpetrator to leave her alone, which was successful in deterring the man from continuing to harass her. From that young researcher: “I really like that [the project director] supported me when I got water thrown on me and I responded in a slightly not-helpful way. [They] gave me helpful feedback about my response and validated me in a way I did not expect.”

Finally, while not a preventative measure, the team conducted a dedicated “debrief” session after each research shift, to discuss what had occurred and provide time for processing stress from the interviews or external entities. One young researcher said: “We were told that there would be some very tough and emotional/mentally taxing moments in the job. And there were. [The project director] did not allow anyone to be attacked and made sure to debrief after moments of tension. Debriefing difficult moments was super helpful.” In addition, throughout the study, the youth researchers were provided with a therapist who conducted weekly group therapy sessions, without any senior researchers present, to ensure they had a safe space to process their experiences in the study.

### Employment and professional development

Two key themes emerged that created challenges for youth researchers to engage in their employment. First, structural barriers specific to YEH made employment difficult from the outset. Second, young researchers had difficulties engaging in consistent professional behavior, such as consistent on-time attendance and proactive communication, as many lacked prior professional experience due to limited opportunities. Additionally, unstable living conditions and a history of basic needs insecurity for some of the young researchers contributed to stress and lack of professionalism.

#### Structural barriers to employment and participation

Youth researchers, especially those exiting or with lived experience of homelessness, faced numerous barriers to employment. This included a lack of access to documentation such as birth certificates, Social Security cards, and photo identification, as well as a lack of a consistent address or bank accounts to which payments could be directed. Several youth researchers struggled with the cost of accessing public transportation, making it more difficult for them to attend work regularly and on-time. Two youth researchers were transgender and were going through the process to change their names on official documentation, which led to stress in receiving paychecks made out to their deadnames (those names assigned to them at birth and not aligned with their current identities). One youth researcher had an active court case from a charge related to the criminalization of homelessness, which led to his incarceration and resulting early departure from the project.

To address these challenges, the senior research team initiated stronger connections between agency administration and the project director. The project director worked closely with administrative leadership from the partner agency to ensure that youth researchers had the information and support they needed to complete employment tasks. The agency additionally provided unlimited passes for public transit to youth researchers to ensure they had consistent access to transportation. The project director provided informal case management to young researchers by connecting them to dedicated case managers and supportive services that could help them obtain documentation or address legal issues as much as possible, while also acting as a contact for case managers who were looking to connect with young researchers who had limited modes of communication, such as phones with few minutes or numbers that changed frequently, and limited access to email and computers.

#### Professional behavior and expectations

Many of the youth researchers had no prior stable, professional employment opportunities or training. In addition, as homelessness and other basic needs insecurities are traumatizing, and many YEH, including the young researchers, have experienced additional traumatic events via interpersonal violence and violence from the police, most of the youth research team exhibited some level of traumatic stress response. Due to these stressors, youth researchers regularly struggled to show up to work on time or to communicate when they were unavailable for work or had scheduling conflicts. These are behaviors that would normally lead to termination in other employment settings. These instances of tardiness and absences, sometimes without notice, created difficulties in the procedure to transit as a group to research sites, as the team would often need to wait for the tardy youth researcher or for confirmation that they would be absent. A young researcher said: “It was really hard for me to make it consistently on time because I just wasn’t used to working a job in a while. I was getting over disability and just leaving the house every day was very traumatic.” Traumatic stressors could also lead to a team member needing to leave their work early for the day, sometimes while in the field conducting interviews. One of the young researchers explained: “Because of life circumstances, because of the way that homelessness functions, and because it’s difficult to keep a job, or to even have any stability at all, it was hard to go to that training; it was hard to keep up with the responsibilities of the job.”

This lack of prior experience and traumatic stress responses contributed to several minor and one major incidents of interpersonal conflict within the group. In the major incident, one of the young researchers was unable to appropriately receive direction from peers and reacted with increasingly disruptive and eventually misogynistic behavior following a data collection shift. This incident led to an extended team meeting of the field staff, facilitated by the project director, that resulted in the group deciding by consensus (including the offending team member) that the team member’s behavior was not conducive to team operations, at which point he left the project. In discussing the incident later, one of the researchers explained: “I think what came up during that conversation and that interaction was that we would defend each other; we will back each other up; we always will defend each other; we’ll always protect each other, because we did. And we all got up and defended each other, and once we experienced that together, and when that sort of hostility was removed from the team, there was a lot more trust. You could see it when we were in the field.” Other minor interpersonal instances were resolved through the debriefing process that was implemented as a safety measure as previously discussed.

To support the young researchers, the project director and senior leadership continually re-oriented daily activities to prioritize trauma-informed practices and communication over the timely completion of the research protocol, balancing structure and flexibility in order to promote capacity-building and strengths-based participation. The team responded to tardy or absent team members with understanding and acceptance, recognizing that each of them struggled on different days. Tasks were oriented to ensure that some work could be completed even while waiting to leave for field operations. The composition of interview teams was actively and flexibly modified, sometimes while already in the field, to ensure that interview teams always had the number of members needed to safely conduct interviews. One researcher described this process: “I learned a lot of coping skills, as far as being outside, because we would have an interviewer and the tech person and the person watching our back. Learning how to navigate was a skill I did not expect to get—just like walking and being conscious of where you are, it was more of a coping skill, a strategy for getting around in the world.”

One of the young researchers explained the value of communication methods: “We would always have multiple ways to communicate, if I did not have a phone or email or anything, and the project updates were always in multiple places. We had an app (GroupMe), we had email, we had text messages, and we had phone calls, so that communication was always there.” Another researcher added: “The communication was extremely helpful, having the app, the phone calls, the text messaging, and the emails. My favorite part was having the weekly schedule. What we would be doing, and the time frame, it was so specific and thought-out beforehand, it was great. I do not know if I would ever have been able to make it this far without that. It’s just like an anxiety relief to know what I’m doing and when I’m doing it.” In another example, a young researcher explained how they were supported in their learning: “There were times when I would kinna panic in the middle of the [practice] interview and [the project director] would notice that and [they]’d be like, ‘Hey, you know, just relax, take a breath …’ you know? [They] made sure that like each of us were able to just step back, take a breath, and get our bearings. And then we could keep going.”

In consultation with the senior study team, the project director engaged in consistent group and individual supervision and mentorship while ensuring that youth investigators were connected to case managers and social services. The project director’s experience with YEH as a clinical social worker and researcher provided a foundation of trauma-responsive professional supervision while ensuring the ongoing completion of research tasks. The experience of unconditional positive regard and “caring” was described as an important mechanism by which the youth researchers were able to feel safe and able to make mistakes. One of the young researchers described this style of supervision: “[They] did really good with handling me in particular because I have a hard time being around people. There were times when I just wasn’t feeling it, like my anxiety was to the point where, I did not wanna leave the house; I could not really do anything, and [the project director] was completely understanding of it.” This researcher added: “there were also times when [they made] sure to check up on me like, ‘Hey, I know this is somewhat of an issue for you, just let me know if you need to take a break, and stand back, or do any of this …’ [They were] very flexible, very patient, I loved the joking, and even the serious times. This is something I wish I could see in every supervisor.” Another young researcher explained: “Whenever I went to the team, into a meeting, into our research, I always felt like I was learning, and that when I made mistakes, you know it wasn’t going to delay the project or ruin it. It was OK. We did a lot—I think part of the structure of our team, and of our meetings, and of our training was that there were kind of these scheduled mistakes, and it was OK to make mistakes, and we did make mistakes.”

### Benefits of participation

In addition to the challenges and opportunities described above, a major theme to emerge was an emphasis on the benefits that youth researchers received from their participation in the study. Youth researchers described multiple benefits, including increased capacity to give and receive feedback, increased perceived self-efficacy in completing a project increased sense of self-worth, and feeling like young people experiencing homelessness are being heard and that “we matter.”

Youth researchers consistently described how the professional environment of the team provided an opportunity for them to safely receive and process feedback. One youth researcher stated “I always felt like when I made mistakes it was not the end of the world.” In addition, youth researchers developed further confidence in themselves by way of their achievements in completing the work of the study, with a youth researcher saying “This is what completing something looks like. I had not really completed a lot of things before this.”

Young researchers described their increased ability to communicate, such as: “just learning how to word things, learning how to present myself and talk to people in a way that gets the job done.” Another young researcher described their growth: “I learned that I needed to work on my patience, it was very difficult for me to maintain a professional attitude with some of the things that we had to deal with, but I also learned I do not really have to be so panicky, I can calm myself better now.” One team member described their change in confidence: “I gained a lot of confidence by multitasking during fieldwork. I was able to pay attention to my surroundings while making sure my work got done.”

Youth researchers pointed to the responsive, accepting, empowering, and inclusive work environment in conducting research as a key attribute of their growth during their participation in the project. They described their opportunities to engage in leadership roles as an empowering aspect of participation. While each youth researcher was trained and able to complete all core research tasks, each team member selected a specialized task to take on as their domain and responsibility. A young researcher explained the effect of the project as a whole: “It restored my faith in humanity because before I started this project, I was having issues going outside. I would sleep a lot because I was suicidal and I would just want to be alone. I did not want to be around people; it wasn’t so much because I hated people, it was just because I got hurt a lot, kind of a sucker because I love everybody, and want to help everybody. [The project] gave me a renewed faith, and it restored my purpose in life, to like help people, and young people, because I never really thought about it before.”

Another young researcher described the independence they felt in the project: “[The project director] was never present at the interviews. It was totally on us to make sure everything worked in the field. I enjoyed that. I enjoyed that a lot. It gave us a lot of room to be accountable and to be responsible, and to really develop our own skills as interviewers. Knowing that we were in charge of pretty much taking this interview from start to finish, I think it allowed us to all become very independent” This researcher added that the project has contributed to their work since, saying: “it gave me the confidence to be able to have conversations with people who are doing important work surrounding health equity and in lowering health disparities.”

## Discussion

Our findings regarding the experiences of young people with lived experience of homelessness as participant-researchers in a study of YEH provide insights into the challenges and opportunities that might emerge within YPAR research with vulnerable youth populations. The findings here, including food and financial insecurity, environmental violence, structural and personal barriers to employment, and the benefits of participation, suggest the need for YPAR researchers to nurture inclusive, trauma-responsive, and flexible environments in which research tasks are completed by participant-researchers.

Food insecurity was an early and prominent issue that emerged in the study and necessitated intentional and creative methods to address. Food insecurity and other basic needs insecurities should be expected in vulnerable youth populations, including YEH as well as other systems-involved young people and youth experiencing poverty.

Participant-researcher compensation has been recognized in the literature and in PAR and YPAR approaches as a notable issue of consideration for ensuring that community partners are respectfully and equitably included in research (11, 35–37). Discourse in the extant PAR/YPAR literature suggests that community partners must be adequately and equally or equitably compensated for their work, in ways that are on par with other research staff and partners (11, 35–37). In our study, youth researchers were hired and paid as agency staff members, completing the same paperwork and engaging in the same employment processes as other agency staff. As marginalized and under-resourced young people, YEH are especially vulnerable to economic scarcity (38). Paying the youth researchers an hourly rate above minimum wage was foundational to our ethical engagement in YPAR research (35, 37).

In light of the economic needs of vulnerable youth, we urge all YPAR researchers to plan for and provide food and financial compensation to their participant-researchers and community co-researchers. Providing food, transportation, and economic compensation are pivotal to ensuring that YPAR participant-researchers can fully engage in the research process. This planning must be included in grant proposals and calls for funding. We recognize that some funders do not allow for food in study budgets, and university policies that disallow the provision of food in many circumstances, which are unfortunate positions considering the issues presented here. These structural limitations further motivate a robust partnership with community agencies, including adequate financial support to the community partner in project budgets in areas that are allowed by funders, which may provide the opportunity for the community partner to cover the cost of food from other sources of funding. In situations where this is not feasible, another possible solution may be the provision of additional compensation to participant-researchers, perhaps as a stipend in addition to wage compensation, with the explicit messaging that such compensation is intended to ensure that they are able to meet their basic needs.

Environmental violence experienced by the young researchers was an unexpected but prominent issue in this study. It is easy for researchers to intellectually understand the environmental risks and vulnerabilities to harassment and violence that are experienced by YEH, but it is another thing entirely to experience those instances of violence, sometimes on a daily basis, within the course of a research study in partnership with young people. Street harassment research has shown that street harassment, unsurprisingly, has negative mental health impacts on victims and witnesses, and is woefully understudied, particularly in relation to perpetration and prevention of street harassment (39). Existing research in social sciences with community-, street-, or field-based approaches have described the ubiquity and concern of sexual harassment of women researchers in fieldwork (40–43), although much of this work has primarily focused on sexual harassment between colleagues and the related power dynamics of gender, supervision, and professional contexts. Literature that does recognize and discuss street harassment of researchers has primarily focused on harassment of women by men, with little attention to-date of homo/transphobic and/or racist harassment of researchers, and with a notable dearth of research on harassment of youth researchers of any identity. YEH at large are more vulnerable to violence than other youth, and this is especially true for LGBTQ youth, women and girls, and youth of color (12, 13, 15, 20). Diversity of research teams and participants is an important component of community-based research, but researchers cannot hire and recruit diverse researchers and research participants without attending to the additional safety concerns that are experienced by marginalized groups (44). Street- or field-based research studies are valuable for engaging participants where they live their lives and can provide important insights, but fieldwork with vulnerable youth requires multiple layers of protection and strategies for preventing and addressing environmental harms.

Our data and experiences do not make clear how much of the environmental violence targeting our young researchers was inherent to conducting research with YEH, a function of street-based walking tour interviews, and/or related to the city in which we operated. Fieldwork with vulnerable youth will likely always carry some risk of environmental violence that needs to be addressed in planning stages and responded to immediately as it occurs throughout the course of a study. YPAR researchers working with vulnerable populations must ensure they are properly prepared and equipped to address environmental harms, including layered safety plans and opportunities for debriefing after each encounter. Our findings that describe environmental violence and street harassment targeting community-based researchers are novel additions to social science research, both in considering prevention and intervention efforts for street harassment in fieldwork broadly and to bring attention to the need for acknowledging and addressing environmental violence experienced by youth researchers.

Structural barriers to employment are a known issue facing YEH and other systems-involved youth, primarily related to discrimination and lack of access to education, healthcare, housing, transportation, and safety that are required for stable employment (23, 45–48). Disrespect and insensitivity by service providers, law enforcement officers, and potential employers is a common experience of YEH, in addition to inadequate services and personal resources, that severely limit opportunities for employment and subsequent financial stability (23, 47–48). In this study, many of those challenges were overcome through consistent communication between research team members and study leadership, in consultation with agency staff and leaders, resulting in quick responses to issues and concerns. The need for case management for the young researchers became clear early in the project and remained a need throughout, necessitating consistent communication within the research teams. The shared leadership of the study and direct communication between academic and agency partners was essential to ensuring that the young researchers could be employed and receive their pay in a timely manner and for providing flexibility and understanding when documentation issues and other challenges arose.

When engaged with YPAR research with vulnerable youth, researchers need to be aware of the potential structural roadblocks that may confront participant-researchers, such as lack of access to transportation, healthcare, food, documentation, and other structural factors. For example, in this study, public transit passes were provided to overcome challenges in transportation. Researchers should seek community guidance to help them plan ahead to address as many challenges as possible in advance to reduce the structural barriers faced by their youth researchers. YPAR researchers need to consider the relationships and understandings between community partners, young participant-researchers, and other staff and youth who are not affiliated with a project. For example, in our study, the young researchers were both staff of the partnership agency (as their employer) and received services from the agency (to address the need for food and case management), which required consistent communication between agency staff affiliated and unaffiliated with the study.

YPAR researchers, especially those working with marginalized youth populations, should be prepared for the traumatic stressors and limited opportunities experienced by their youth researchers. It can be difficult for youth to arrive at work on time, especially those navigating public transportation and in unstable living conditions, or to provide notice if they will be late or absent, which is particularly relevant to youth without phones or whose phones have limited minutes or data. YPAR researchers should be prepared to witness traumatic stress responses, including impulsivity, emotionality, physical and mental health concerns, hypervigilance, and other expressions of trauma (2, 28, 49, 50). A trauma-responsive approach that is grounded in unconditional positive regard is fundamental to successful YPAR with vulnerable youth (28, 51–54). That is not to say that youth should not be held accountable for their behavior, but it does mean that behaviors that are not harmful to others should be addressed with gentle correction and constant flexibility. It is vital to foster an attitude of care as an aspect of mentorship and team-building within YPAR projects (2, 28, 50, 52, 55). YPAR researchers need to intentionally create an environment of inclusion and unconditional positive regard, allowing youth researchers to grow and make mistakes and be safe in doing so (50).

The benefits described by the young researchers in reflection on their experiences with the study was an important finding in this study. The young researchers developed leadership and communication skills in a safe environment that allowed them to make mistakes and take on responsibilities that encouraged their growth. By recognizing the importance of youth voice in the foundational philosophies of the research itself, young people were able to use their skills and experiences to inform policies, services, and resources for young people in similar life circumstances. This focus on empowerment and growth is the reason YPAR exists and must be recognized as central and necessary to the success of any research that involves young people and connects with their communities.

This study demonstrated several key successes of YPAR approaches by providing opportunities for participant-researchers to: increase self-awareness, develop critical consciousness on power dynamics and systems, and provide empowerment through leadership roles and task completion (3, 4). YPAR research requires a focus on the approach to research as itself a benefit to youth researchers (44, 56) and this focus necessitates safe space in order for youth researchers to access and engage in beneficial experiences (2, 50). By providing job experience, opportunities for improving their own communities, and the time and space for developing skills and insights, YPAR can improve the lives of youth researchers through the very act of conducting research itself (3, 4, 50, 56). In addition, peer engagement has been shown to improve outcomes for youth communities, and youth researchers in this study made clear that their wellbeing was improved by having the opportunity to provide support to peers and their communities (29, 51).

Communication is a critical mechanism by which the issues and needs described in this study were identified and resolved. By ensuring consistent and open communication between youth researchers and the senior research team and the community partner agency, many of the issues that affected the youth researchers were able to be addressed quickly and effectively. This approach to open communication provided youth researchers with the attention and engagement they needed to effectively implement research tasks and grow as professionals. Effective communication required that the senior research team prioritized the wellbeing of youth researchers over the efficient completion of research tasks, which was a foundational philosophy of the senior research team from the beginning of the partnership that led to this YPAR study. YPAR researchers must follow similar approaches to prioritize the wellbeing of youth researchers, communicating internally and with external partners to adapt to situations that will inevitably arise and impact the lives of vulnerable youth researchers.

### Strengths and limitations

There are notable limitations to this study with this vulnerable group of young people. The small sample of six participant-researchers suggests the need for more research that attends to similar ideas explored here for confirmation of these findings. The challenges and vulnerabilities experienced by this group may not be as relevant or prominent in other YPAR settings, due to the particular nature of urban street-based research initiatives, in addition to the particular needs of YEH. Nevertheless, YPAR researchers should be attentive to the particular needs of the youth populations with whom they engage. Young people are vulnerable to systemic and environmental stressors; youth from marginalized backgrounds even more so. As previously mentioned, the risks of environmental violence and structural barriers in conducting research are likely attributable to the inherent risks of conducting research with YEH, conducting research using street-based methods, or conducting research in dense urban areas, or likely some combination of the above. Our experiences and responses may have been specific to our study contexts and less relevant to YPAR with different youth populations, methods, and/or settings.

A strength of this study was the strong partnership between the academic researchers and social service agency leaders who comprised the senior research team. The funding provided for the study required that the research team be led by academics and community partners. This relationship was critical to the success in responding to challenges in the project. The study teams communicated regularly to respond to challenges that emerged almost daily at certain points in the study. The partner agency’s commitment to trauma-responsive values directly contributed to the flexibility in providing resources and services to meet the needs of the youth researchers. Without that partnership, this study would not have been able to successfully support the young researchers. YPAR researchers should be intentional in developing strong partnerships with community agencies that are committed to supporting research and which have access to services to address the inevitable and legitimate needs of young researchers. Finally, the project director’s combined experience as a clinical social worker and researcher with experience conducting fieldwork with YEH was a critical factor in the success of recognizing and implementing effective responses to the needs of young researchers. This experience suggests that it is valuable to have a research team with direct experience and expertise with the population of study, both in providing care and services and in conducting research in a given setting and context.

## Implications and conclusion

Basic needs insecurity and homelessness are destabilizing factors that limit the opportunities for young people to engage in professional settings, necessitating intentional and collaborative efforts on the part of academic researchers to support youth researchers so they may engage meaningfully in YPAR. Researchers engaged in youth-centered participatory research must accept the obligation to support youth researchers in ensuring their basic needs during participation in YPAR research. When planning YPAR research, scholars should budget and plan for contributions and connections to ensure that youth research participants are adequately fed, hydrated, housed, and safe. Participatory- and community-based research with young people experiencing homelessness must flexibly adapt projects to support the basic and complex needs of the young people involved in the research process by developing intentional, trauma-informed approaches to empower participation of young researchers without putting them at further risk of harm. Clear and consistent communication is a key mechanism by which issues can be identified, examined, and addressed during a study, ensuring that the needs of youth researchers can be supported as quickly, effectively, and inclusively as possible.

Action is a central component of YPAR and is a foundational goal in the implementation and motivation for engaging in this work. The emphasis on action in YPAR is often focused on external changes and how community-engagement with research can contribute to wider changes in policies, services, and programs. The wider project in which this study emerged has been utilized to contribute to these changes (see [authors blinded], 2023 and [authors blinded], 2023). The study presented here suggests there can be, and needs to be, action on the part of researchers themselves during the course of a YPAR study with vulnerable youth. Actions were needed and taken to ensure that young researchers had adequate food, had avenues to prevent and address environmental violence, and were offered inclusive, affirming supervision in their work. The action of YPAR can support growth in youth researchers, to the benefit of their self-esteem, confidence, professional experiences, and opportunities for relationship-building.

## Data availability statement

The datasets presented in this article are not readily available because authors do not have permission to share datasets directly, due to the consent process in collecting these qualitative data. Requests to access the datasets should be directed to GAR, aratliff@unr.edu.

## Ethics statement

The studies involving humans were approved by the Committee for Protection of Human Subjects at the University of California, Berkeley. The studies were conducted in accordance with the local legislation and institutional requirements. The ethics committee/institutional review board waived the requirement of written informed consent for participation from the participants or the participants' legal guardians/next of kin because written consent would have been the only collected identifiable data and therefore increase the risk of harm in case of any unintended breach of confidentiality.

## Author contributions

GAR: Conceptualization, Data curation, Formal analysis, Investigation, Methodology, Project administration, Resources, Software, Supervision, Validation, Writing – original draft, Writing – review & editing. DC: Formal analysis, Validation, Writing – original draft, Writing – review & editing. JY: Data curation, Formal analysis, Investigation, Methodology, Project administration, Software, Validation, Writing – original draft, Writing – review & editing. RS: Data curation, Formal analysis, Investigation, Methodology, Project administration, Software, Validation, Visualization, Writing – original draft, Writing – review & editing. TH: Data curation, Formal analysis, Investigation, Methodology, Project administration, Software, Validation, Writing – original draft, Writing – review & editing. NJ: Formal analysis, Investigation, Methodology, Writing – original draft, Writing – review & editing. ML: Conceptualization, Data curation, Funding acquisition, Investigation, Methodology, Project administration, Resources, Supervision, Validation, Writing – original draft, Writing – review & editing. SA: Conceptualization, Funding acquisition, Investigation, Resources, Supervision, Writing – original draft, Writing – review & editing. IL: Conceptualization, Funding acquisition, Investigation, Resources, Supervision, Writing – original draft, Writing – review & editing. CA: Conceptualization, Data curation, Formal analysis, Funding acquisition, Investigation, Methodology, Project administration, Resources, Software, Supervision, Validation, Visualization, Writing – original draft, Writing – review & editing.
